# A process monitoring microreactor assembly for real-time reaction analysis using inline near-infrared spectroscopy and chemometrics

**DOI:** 10.1007/s00216-025-05779-2

**Published:** 2025-02-07

**Authors:** Lukas Mahler, Pascal Desel, Marcel Sladkov, Andreas Roppertz, Christian Mayer, Martin Jaeger

**Affiliations:** 1 Department of Physical Chemistry, University Duisburg-Essen, Essen, Germany; 2 Department of Chemistry and ILOC, Niederrhein University of Applied Sciences, Krefeld, Germany

**Keywords:** In-line monitoring, Process analytical technologies, Online NIR, Platform chemicals, Real time

## Abstract

The occurrence of highly exothermic and potential runaway reactions represents a significant hazard to workers, facilities, and the surrounding ecosystem. Due to their high reaction velocity, these reactions often prove difficult to monitor. In many instances, the recorded data result from several single-quantity sensors, such as temperature and pressure sensors. Yet, deviations from the anticipated process, and thus potentially hazardous reaction parameters, may only be identified by the combination of the sensor data, leaving the cause of the deviations unexplained. In this study, a custom-built microreactor in combination with in-line near-infrared spectroscopy and off-line gas chromatography was employed to allow monitoring of the acid-catalyzed esterification of acetic acid and methanol. Using a near-infrared spectrometer in conjunction with partial least squares regression models, reactant and product concentrations could be determined at various residence times and temperatures. This approach enabled the safe and efficient investigation of previously unexamined parameter ranges and the expansion of the design space of experiments. The near-infrared spectra were subjected to principal component analysis classification in order to obtain additional qualitative information. The conversions and space–time yields of the microreactor could be calculated from the obtained data. The values indicated that the microreactor design delivered yields up to twice as high as those of commonly used batch reactors. This study hence demonstrates the efficacy of spectroscopic methods for reaction monitoring in microreactors and their potential for application in micro process analytical technologies.

**Graphical Abstract:**

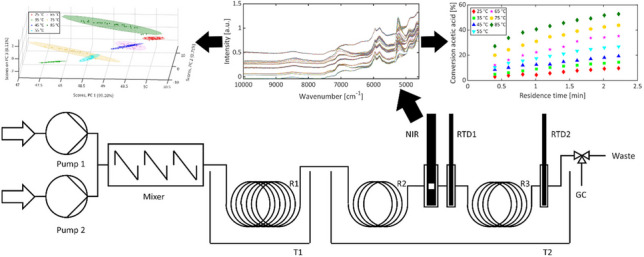

**Supplementary Information:**

The online version contains supplementary material available at 10.1007/s00216-025-05779-2.

## Introduction

In the field of chemical engineering and process optimization, process analytical technologies play an important role today. The capacity to monitor and subsequently regulate reactions in real time is of paramount importance. The necessity for the efficient utilization of supplies, process safety, and quality enhancement requires the implementation of rapid and reliable analytical methods for the monitoring of production processes [1, 2]. In an ideal scenario, real-time monitoring would facilitate the identification of deviations from an anticipated process progression [3, 4]. Based on the real-time data and process models, control measures can be implemented to return the process to the desired specifications [5, 6]. This is of particular importance for potentially hazardous reactions that are highly exothermic and/or prone to runaway conditions. The common methodology for their investigation employs calorimeters. These calorimeters quantify the total amount of heat released by the reaction, yet they lack the capacity to discern between simultaneous initiation in a homogeneous reaction mixture and hotspot initiation [7, 8]. Single-quantity sensors, such as pH meters, gas sensors, and resistance temperature devices, provide real-time data, but offer limited information content. In contrast, spectroscopic methods provide multi-component information [9, 10]. They allow much faster measurement times than their chromatographic counterparts, such as gas chromatography (GC) [11]. The multi-component information typically yielded by spectroscopic monitoring allows for the tracing of reactants, products, and, depending on the sensitivity of the method employed, by-products simultaneously. In instances where the data is highly complex, such as in crowded spectra, the employment of chemometric methods or multivariate data analysis techniques is typically necessary in order to simultaneously harness all variables. Regression methods in multivariate data analysis techniques, such as partial least squares regression (PLS-R), are employed to determine concentrations from spectra. This approach allows for the extraction of the most pertinent information from the data and its correlation with a calibration [12, 13]. Principal component analysis (PCA) is a widely utilized non-regression, qualitative multivariate data analysis technique that is effective in identifying similarities and differences between data series, such as spectra. Although it is not suitable for determining concentrations or other quantitative variables, it can nevertheless be employed for process monitoring in a semi-quantitative or classification manner [14–16]. Concurrently, near-infrared (NIR) spectroscopy established itself as a powerful tool for real-time reaction monitoring in various industrial applications [17]. It is known for its non-destructive and rapid analytical capabilities, which makes it an invaluable technique in many application fields. The data it produces are often highly complex, yet they can ascertain a wealth of information [4, 18, 19].

Microreactor technology has the potential to transform the methodology for studying runaway reactions, offering enhanced safety, through a reduction in reaction volumes, optimized heat and mass transfer, and precise control over reaction parameters [20–22]. These reactors are constructed from a variety of materials, including silicon, polymers, glass, and metal, and can be designed as capillary or chip reactors [23–26]. Nevertheless, the combination of in-line spectroscopic methods with microreactors for real-time reaction monitoring remains a powerful and promising approach, offering a significant opportunity for advancing the understanding and control of complex chemical processes [18, 27–29]. The esterification of acetic acid and methanol, catalyzed by sulfuric acid, represents an excellent model reaction for investigating the potential of combined microreactor and in-line spectroscopic technologies. While the reaction is well-understood, its characteristics include the sensitivity to temperature and reactant ratios, as well as the potential for high conversion rates [30–32]. The spectroscopic approach in a microreactor setup allows for the monitoring and investigation of reaction conditions that were previously deemed too dangerous or impractical to examine [33].

In this study, a custom-built reaction monitoring assembly that integrates in-line NIR spectroscopy and off-line gas chromatography served for the observation of the esterification of acetic acid and methanol catalyzed by sulfuric acid. Real-time observation was accomplished to allow precise control of reaction conditions. The reaction was examined at various residence times and temperatures. The NIR spectra recorded during the reaction were utilized for quantification by PLS-R and classification by PCA at different reaction dynamics. Based on the concentrations determined by in-line NIR spectroscopy and off-line gas chromatography, conversions and space–time yields were calculated and compared to those of commonly used batch reactors.

## Material and methods

### Reaction monitoring assembly

**Fig. 1:**
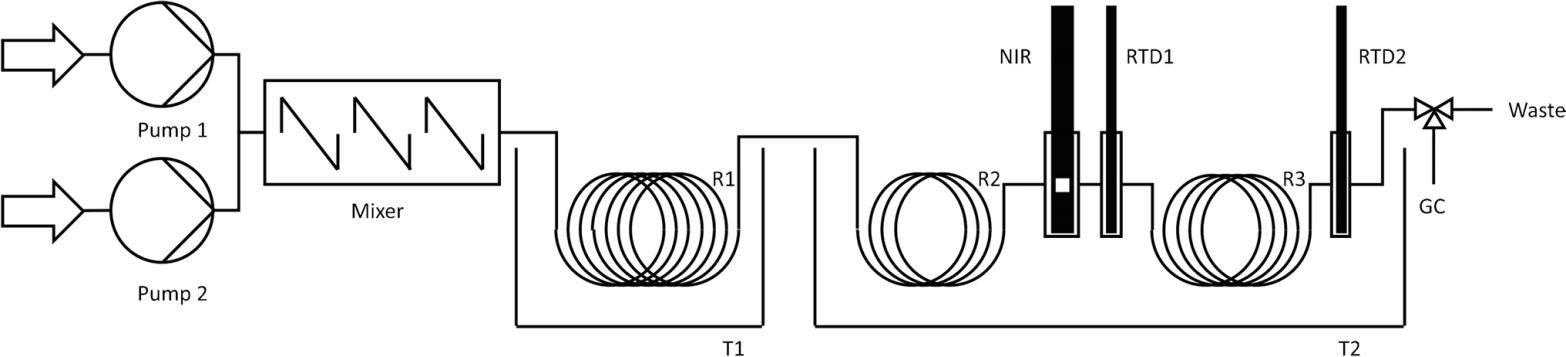
Reaction monitoring assembly with two pumps for the reactants, mixer, water baths (T1, T2), reactors (R1, R2, R3), resistance temperature devices (RTD1, RTD2), together with NIR probe for spectroscopic analysis and valve for collecting the samples for off-line gas chromatography (GC) of the acid-catalyzed esterification of acetic acid and methanol

Two Smartline pumps (KNAUER Wissenschaftliche Geräte GmbH, Berlin, Germany) conveyed the reactants into a micro-mixer LH2 (Ehrfeld Mikrotechnik GmbH, Wendelsheim, Germany) with a slit width of 25 µm, as illustrated in Fig. 1. Subsequently, the combined reactants flowed through the first microreactor (R1), a 5-m-long stainless steel capillary reactor with an inner diameter of 1 mm and an outer diameter of 2 mm. This integral reactor for initial reaction conversions was placed inside a water bath that was controlled via the thermostat ministat 230 (Peter Huber Kältemaschinenbau SE, Offenburg, Germany) (T1). The reaction mixture was further conveyed through a second microreactor (R2), which was another capillary reactor of identical radial dimensions and a length of 50 cm, to ensure isothermal conditions. A third microreactor (R3) with the same radial dimensions and a length of 1 m was situated behind R2. In order to observe the state of the reaction mixture at the inlet of R3, a NIR transflection immersion probe Falcata Lab (Hellma GmbH & Co. KG, Muellheim, Germany) with an external diameter of 6 mm and a cell diameter of 1 mm was embedded in a module made of stainless steel (see Fig. S1) and positioned between R2 and R3. This module was designed to minimize dead volume, thereby eliminating systematic errors. Prior to and following the differential reactor R3, temperature measurements were obtained of the reaction mixture using type Pt100 resistance temperature devices (RTD1 and RTD2) with external diameters of 1.5 mm and a length of 100 mm in combination with an ALMEMO® 710 data logger (Ahlborn Mess- und Regelungstechnik GmbH, Holzkirchen, Germany). The resistance temperature devices were positioned within dead volume minimizing modules made of stainless steel with an internal diameter of 2.5 mm that ensured the measurement of the reaction mixture without touching the stainless steel assembly (see Fig. S1). The reactors R2 and R3, the NIR module, and both resistance temperature devices were situated within a second thermostat ministate 230 controlled water bath (T2) in order to ensure isothermal conditions. Subsequently, a valve for collecting the samples at the exit of R3 for off-line gas chromatography, a backpressure regulator set to 3 bar, and the waste container was placed outside the water bath T2.

### Spectral recording and processing

Near-infrared spectra were recorded inline using an Antaris II FT-NIR Analyzer (Thermo Fisher Scientific Inc., Madison, WI, USA) equipped with a NIR transflection immersion probe Falcata Lab with a path length of 2 mm. The NIR spectrometer was controlled using Result 3 SP7 Build 16 software (Thermo Fisher Scientific Inc., Madison, WI, USA). The spectral range was set from 4600 to 10,000 cm^−1^ with a resolution of 4 cm^−1^ and 32 scans per spectrum. Each spectrum consisted of 1402 data points. The optimized preprocessing of the spectra was achieved through the implementation of 0th order Savitzky-Golay smoothing with a filter width of three points [34], standard normal variate transformation, and baseline correction using MATLAB R2021a Update 7 (MathWorks, Inc., Natick, MA, USA).

In order to obtain samples for the calibration of the NIR models, the differential reactor R3 and the two resistance temperature devices were disconnected from the reaction assembly. The disassembly ensured that the samples were obtained without delay following their measurement with the NIR spectrometer. Subsequently, the samples were quenched with 80 µl of ammonia (32%, Bernd Kraft GmbH, Duisburg, Germany), stored in an ice bath, and analyzed via off-line gas chromatography. By varying the temperature between 25 and 85 °C and the combined volumetric flow rates of the two reactants between 3.13 and 17.20 ml/min, representing residence times between 105 and 19 s, a total of 28 different reaction states were covered, and ranges of concentrations for reagents and products were achieved (*cf.* Table S1). To ensure steady-state conditions, 3 NIR measurements were conducted for each of the 28 calibration samples over a 4-min period, resulting in a total of 84 NIR spectra (*cf.* Fig. S2), which were utilized together with the concentrations determined by GC to build one PLS1 model for each component using PLS_Toolbox 9.0 (Eigenvector Research, Inc., Manson, WA, USA). The spectral data and their associated concentrations derived from the gas chromatographic analyses were divided into calibration (56 samples) and validation (28 samples) data sets using the Mahalanobis distance. Venetian blinds with 10 data splits and one sample per blind were employed as cross-validation method. The quality of each model was evaluated by comparing the predicted concentrations of the validation data set to the measured concentrations. The deviation of the mathematical fits from the theoretically expected angle bisection was further assessed as shown in Fig. S3. Additionally, the root of the mean square error sum of the prediction (RMSEP) was taken into account (*cf*. Table S2). This value was calculated as usual (*cf*. Eq. S1).

### Chromatographic separation and processing

The quantitative reference determinations of methanol, acetic acid, methyl acetate, and water were conducted off-line using a gas chromatograph 8860 GC system (Agilent Technologies, Inc., Santa Clara, CA, USA) equipped with a HP-PLOT U column (Agilent Technologies, Inc., Santa Clara, CA, USA) with dimensions of 30 m × 0.32 mm inner diameter and a film thickness of 10 µm. The carrier gas was helium (≥ 99.999%, Messer Industriegase GmbH, Bad Soden, Germany) with a linear velocity of 32 cm/s. The inlet temperature was set to 200 °C. A volume of 1 µl of each sample was injected with a split ratio of 100:1. Every sample was analyzed three times. The temperature of the column oven commenced at 50 °C and increased at a rate of 15 K/min to a final temperature of 170 °C, which was maintained for a period of 10 min. This resulted in a run time of 18 min. The system was equipped with a thermal conductivity detector set to 200 °C with a reference flow of 10 ml/min of helium and a makeup flow of 5 ml/min of helium. The detector exhibited a signal frequency of 5 Hz. The interpretation of the chromatograms was achieved via OpenLab CDS 2.7 software (Agilent Technologies, Inc., Santa Clara, CA, USA). Calibration plots and formulas are given in Fig. S4 and Table S3.

### Sulfuric acid-catalyzed esterification of acetic acid and methanol

For the examination of the acid-catalyzed esterification of acetic acid and methanol, acetic acid (100%, Carl Roth GmbH & Co. KG, Karlsruhe, Germany) and methanol (≥ 99.9%, Carl Roth GmbH & Co. KG, Karlsruhe, Germany), mixed with 60 mol/m^3^ of the catalyst sulfuric acid (96%, Bernd Kraft GmbH, Duisburg, Germany), were combined via the two pumps and the mixer. The pump speeds were set to ensure an equimolar ratio of the two reactants in the reaction monitoring assembly. The pump speeds were systematically varied between 3.13 and 17.20 ml/min. This resulted in combined residence times for R1 and R2 between 82.88 and 15.07 s and between 15.07 and 2.74 s for R3. The combined internal volume of R1 and R2 was 4.32 ml, and the internal volume of R3 was 0.79 ml. Furthermore, the temperatures at T1 and T2 were altered coherently between 25 and 85 °C, with increments of 10 K, resulting in 70 distinct states of the reaction mixture. To ensure that the conditions remained constant throughout the experiment, 3 NIR measurements were recorded over a 4-min period for each experiment. Following evaluation, 4 experiments were repeated, resulting in the collection of 222 NIR spectra between reactors R2 and R3. The concentrations of the 4 components, acetic acid, methanol, methyl acetate, and water, at the inlet of microreactor R3 were calculated using the previously built NIR models. To determine the conversions in microreactor R3, samples were obtained at the outlet of the reactor for analysis via GC.

### Principal component analysis

The preprocessed NIR spectra were subjected to principal component analysis (PCA) using PLS_Toolbox [35]. The resulting scores were plotted along the first three principal components (PCs) to illustrate the changes in the spectra during each reaction temperature and to evaluate the uniformity of the recorded spectra. Additionally, the scores of the second principal component were plotted against the sample numbers to expose the coherence between the scores and the temperature or the conversion, respectively.

### Conversion and space–time yield

The conversion of acetic acid ${X}_{AcOH}$ was calculated according to Eq. 1 [36]:

1 $${X}_{AcOH}\left(t=t\right)=\frac{{N}_{AcOH,t=0}-{N}_{AcOH,t=t}}{{N}_{AcOH,t=0}}=\frac{{\dot{n}}_{AcOH,t=0}-{\dot{n}}_{AcOH,t=t}}{{\dot{n}}_{AcOH,t=0}}=1-\frac{{C}_{AcOH,t=t}}{{C}_{AcOH,t=0}}$$

where ${N}_{AcOH,t=0}$ is the molar amount of acetic acid at the beginning of the reaction, ${N}_{AcOH,t=t}$ is the molar amount of acetic acid at a time $t=t$, and ${\dot{n}}_{AcOH,t=0}$ and ${\dot{n}}_{AcOH,t=t}$ are the molar flows of acetic acid at the beginning and at a time $t=t$, respectively. The concentrations of acetic acid at the beginning and at a specific time $t=t$ are represented by ${C}_{AcOH,t=t}$ and ${C}_{AcOH,t=0}$, respectively.

In the case of the investigated reaction, yield and conversion corresponded to the same value, due to the absence of side or follow-up products in the reaction regime. The conversion was considered a direct measure for the production of methyl acetate, the main product.

The space–time yield (*STY*) was calculated based on the NIR and GC data and reaction (Eq. 2):

2 $$\text{MeOH}+\text{AcOH}\rightleftharpoons \text{MeOAc}+\text{H}{}_{2}\text{O}$$

The *STY* was used for the comparison of different reactors and was defined according to Eq. 3 [37].

3 $$STY=\frac{{\dot{m}}_{MeOAc}}{{V}_{R}}$$

where ${\dot{m}}_{MeOAc}$ is the resulting product per hour and ${V}_{R}$ is the corresponding reactor volume.

## Results and discussion

**Fig. 2:**
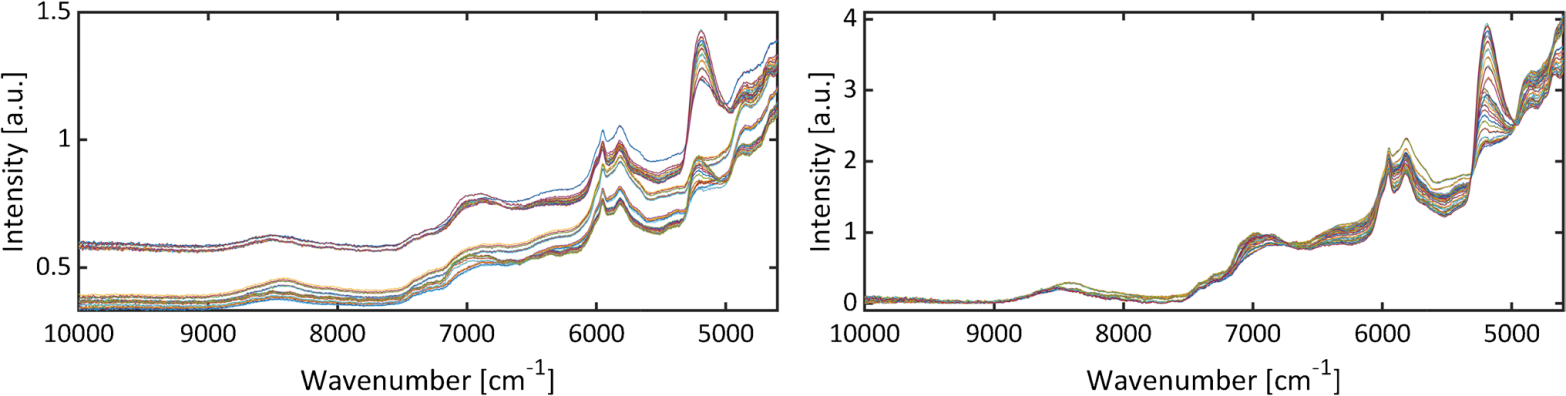
222 NIR spectra of the esterification of acetic acid and methanol, catalyzed by sulfuric acid, with different reaction temperatures and residence times, before (left) and after (right) preprocessing via 0th order Savitzky-Golay smoothing with a filter width of three points, standard normal variate transformation, and baseline correction

The esterification was monitored using in-line NIR spectroscopy. The resulting NIR spectra exhibited disparate baseline offsets, which depended on the temperature and the flow velocity. Hence, preprocessing of the spectra was required, resulting in homogeneous data sets (*cf.* Figure 2) which allowed to extract the critical process parameters at the inlet of microreactor R3. From NIR spectra and concentration data obtained from GC analysis, PLS1 models were established for reactants and the product, such that actual concentrations could be determined from in-line NIR monitoring. These actual concentrations combined with off-line GC analysis at the outlet of R3 yielded the conversions in the microreactor and the space–time yield of the reactor according to Eqs. 1 and 3.

### Conversion and space–time yield

**Fig. 3:**
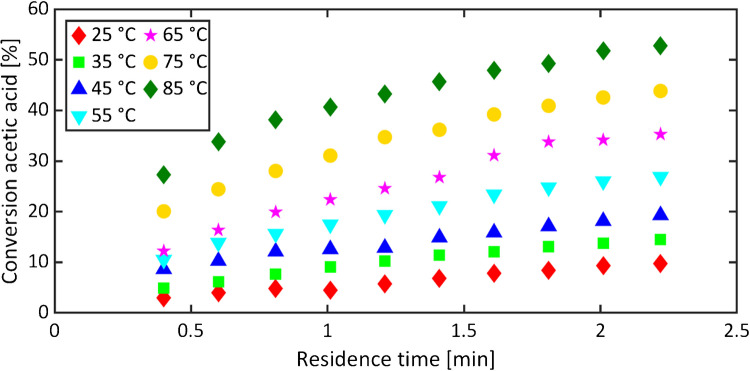
Conversions of acetic acid at the end of microreactor R3 plotted against the residence time at different reaction temperatures

Conversion and *STY* diagrams illustrate the progression of the reaction and facilitate a comparative analysis of the microreactor assembly with other reactors. The conversions were calculated according to Eq. 1. Figure 3 shows the conversions for the microreactor R3. Acetic acid conversion increased with residence time. A prolongation of 2 min approximately doubled the conversion. Higher temperatures led to higher conversions, as reaction rates increase with temperature in accordance with Arrhenius’ law.

Brief reaction times of up to 2.2 min were monitored, yet achieving conversions of up to 53% at 85 °C. The space–time yields for different temperatures were calculated according to Eq. 3 and compared with values reported for batch reactors [38, 39].

**Fig. 4:**
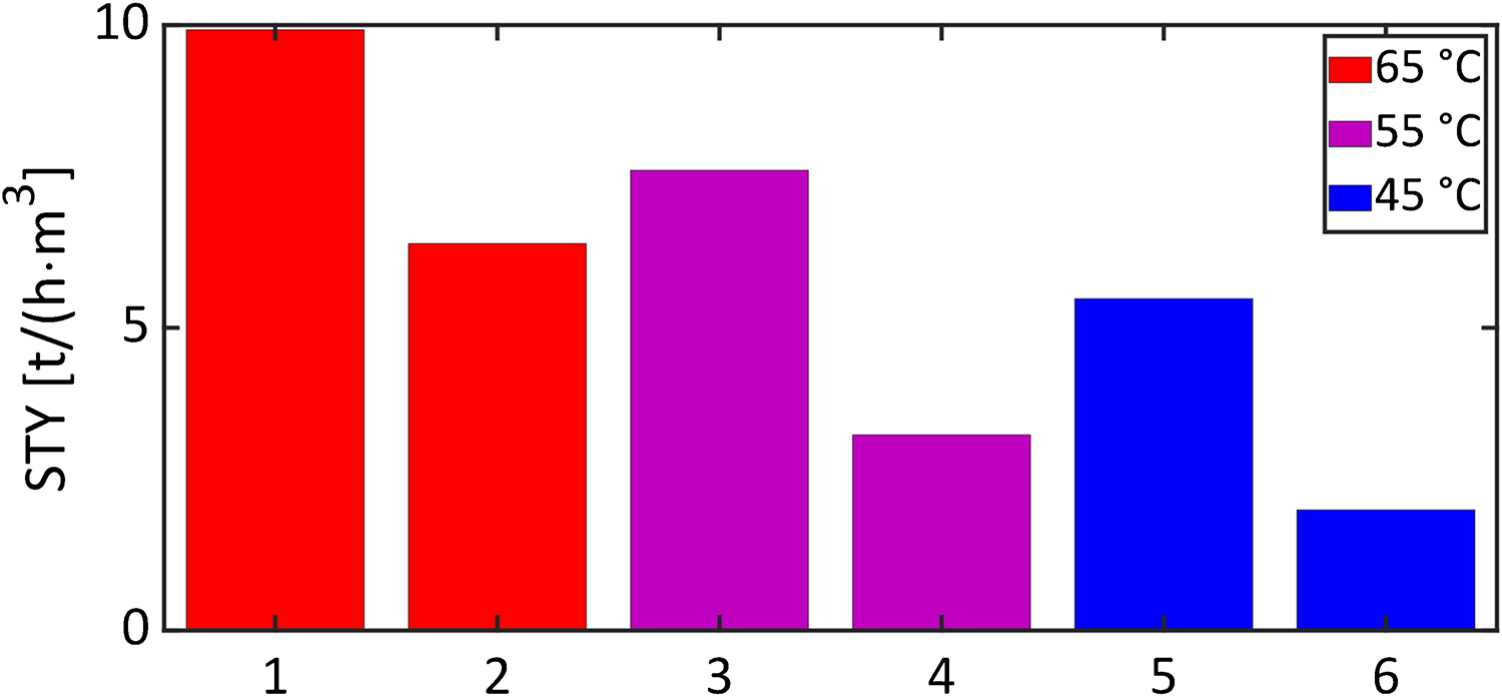
Space-time yields of the microreactor system (1, 3, and 5) and a 500 ml batch reactor (2, 4, and 6) with 89 mol/m³ catalyst at different temperatures. The values of the 500 ml batch reactor were taken from [39], calculated with the total reactor volume used for production and without change-over times

Figure 4 illustrates that the microreactor assembly produced up to 2.8 times the amount of methyl acetate in comparison to conventional batch reactors, even when operated at identical temperatures and catalyst concentrations. The change-over times of the batch reactors were excluded from the calculation, which yields the best-case scenario for the batch reactors. A more rigorous comparison between the microreactor system and the batch reactors is achieved by taking the effective rate constants at different temperatures into account (cf. Table 1). The rate constants of the microreactor system were found between 1.7 and 2.3 times higher than those of the batch reactors. The superior performance may be attributed to the more effective mass and heat transfer in microreactors. Additionally, the homogeneous distribution of the catalyst within the reaction media, which was achieved by using the Ehrfeld micro-mixer LH2, was assumed to contribute to the enhanced production. This properties have made microreactors a highly attractive assembly for the intensification of chemical reactions, as their higher production and heat transfer rates make them safer and more efficient for production purposes [23, 26, 28, 29, 40].

**Table 1:** Effective reaction rate constants of the esterification of methanol and acetic acid, based on the microreactor system (MRS) and [39]

Reactor size	Temperature	Catalyst concentration^a^	k_eff_^b^
MRS	55 °C	89	0.02508
500 ml	55 °C	89	0.01110 [39]
MRS	65 °C	89	0.03742
500 ml	65 °C	89	0.02190 [39]

^a^ [mol/m³]; ^b^ [l/(min∙mol)]

### Principal component analysis

**Fig. 5:**
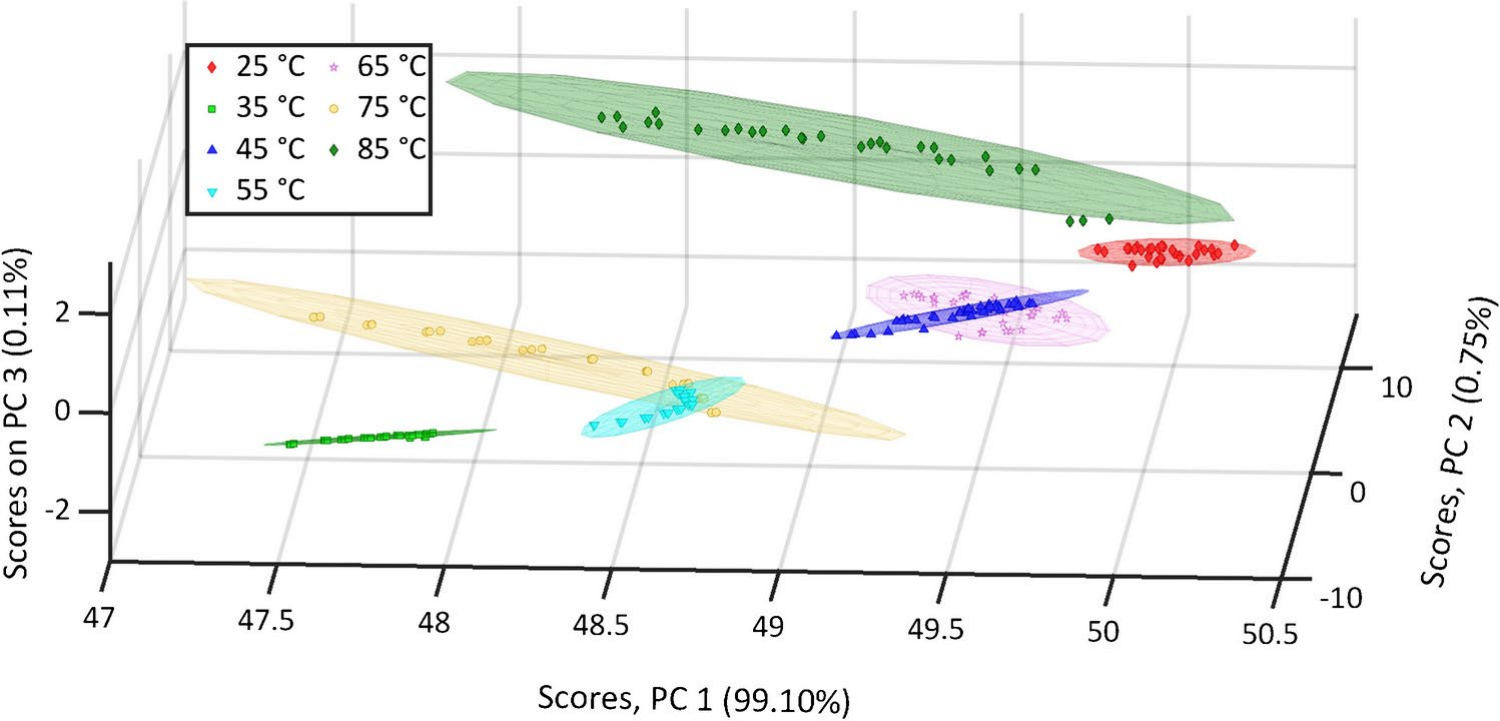
Scores plot of PC 1 vs. PC 2 vs. PC 3 of the 222 NIR spectra categorized in the seven different reaction temperatures with their individual confidence ellipses

To gain further insight into the esterification process, PCA was applied. To accommodate the variation in the data, three principal components proved sufficient. Their three-dimensional visualization is shown in Fig. 5. Their two-dimensional equivalents are given in Fig. S5, while the loadings of the principle components are given in Fig. S6.

In 3D space, the confidence ellipses encircling each reaction temperature were non-overlapping (*cf.* Figure 5 and Fig. S5). Therefore, each reaction temperature could be distinguished from the position of its NIR spectrum scores. Moreover, the degree of conversion at a specific reaction temperature could be derived. The scores of the spectra recorded at 25 °C exhibited a slight spread, indicating minimal spectral variation and thus sparse conversion, which was to be expected at moderate reaction temperatures. In contrast, the scores of the spectra recorded at 85 °C exhibited a considerable spread, indicating a notable spectral variation and thus a substantial conversion. Furthermore, the dispersion of the spectra for each residence time could be evaluated. The spectra of the reaction temperatures 35 °C, 55 °C, 75 °C, and 85 °C displayed the least dispersion, indicating spectral uniformity. In contrast, the spectra of the reaction temperatures 25 °C, 45 °C, and 65 °C exhibited greater dispersion. A more detailed inspection of Fig. 5 revealed a slight inconsistency in PC 3 observed between the scores of the spectra at a specific residence time during the reaction at 85 °C. This deviation was also apparent in the spectra (*cf.* Fig. S7) but was not considered significant. This example demonstrates the utility of PCA as a method for identifying divergent data. In a well-characterized system with validated models, these findings can prompt further investigation to determine whether the recorded spectra or the reaction itself were the source of the deviation.

**Fig. 6:**
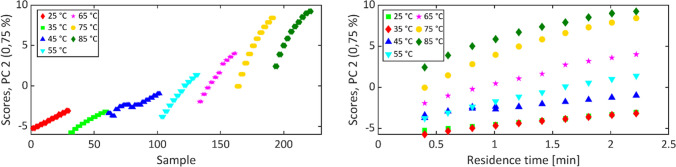
Scores plot of PC 2 vs sample numbers (left) and vs residence time (right) of 222 NIR spectra categorized in the seven different reaction temperatures

In more detail, the influence of the reaction temperature on the conversion could be made evident along PC 2. In Fig. 6, the scores of the spectra are plotted on PC 2 versus the sample number and the residence time, which reflects reaction progress at a given temperature and residence time.

A direct correlation was therefore obtained between the reaction temperature and the spread of the scores, indicating spectral variations and, thus, a conversion variation. As illustrated by the graphs, the data clearly demonstrated that lower temperatures and lower residence times resulted in reduced conversion, whereas higher temperatures and higher residence times yielded enhanced conversion. Additionally, Fig. 6 illustrates an anomalous behavior of the spectra recorded at 45 °C. While the scores on PC 2 increased with residence time at all temperatures, some scores of the spectra for 45 °C appeared to decline with increasing sample numbers. Upon inspection of the corresponding NIR spectra (*cf.* Fig. S7), it became evident that this finding could not be substantiated. Consequently, the data were discarded, and the experiment was repeated. This resulted in the anticipated outcome of increased PC 2 scores. Subsequent investigation revealed that the cause of this outlier was an improperly tempered water bath.

In summary, PCA visualizes the reaction course as well as deviations in a non-quantitative manner, i.e., concentrations. As data preprocessing and multivariate data analysis can be conducted in an automated manner and a very short time, this approach may be employed to detect and identify process and spectral deviations in well-established processes in real time. The method would also be beneficial when calibrations are exceedingly time-consuming and precise knowledge of the concentrations is neither crucial nor available.

## Conclusions

In this study, a custom-built reaction monitoring assembly was examined for the purpose of monitoring the esterification of acetic acid and methanol catalyzed by sulfuric acid through the use of in-line NIR spectroscopy and off-line gas chromatography. It was demonstrated that the implementation of real-time NIR spectroscopy in combination with multivariate data analysis facilitated the determination of conversions, space–time yields, and effective reaction rate constants. These estimates could be achieved while operating within process parameters that would result in critical conditions within batch reactors. Additionally, the classification of NIR spectra via PCA was feasible, and invalid data or reaction deviations could be identified. The microreactor assembly was proven to yield space–time yields and effective reaction rate constants superior to those of commonly used batch reactors. As the assembly allows to monitor reactions that are highly exothermic and potentially hazardous, it has the potential to enhance their safety and efficiency optimization. Real-time monitoring could offer a powerful tool for expanding the design space of experiments to encompass previously unexamined parameter ranges. Moreover, a comprehensive understanding of reaction behavior, obtained on a microscale, can facilitate the development of more cost-effective and environmentally friendly processes.

## Supplementary Information

Below is the link to the electronic supplementary material.

Supplementary file1 (DOCX 1078 KB)
