# Correction to: A process monitoring microreactor assembly for real-time reaction analysis using inline near-infrared spectroscopy and chemometrics

**DOI:** 10.1007/s00216-025-05937-6

**Published:** 2025-06-02

**Authors:** Lukas Mahler, Pascal Desel, Marcel Sladkov, Andreas Roppertz, Christian Mayer, Martin Jaeger

**Affiliations:** 1 Department of Physical Chemistry, University Duisburg-Essen, Essen, Germany; 2 Department of Chemistry and ILOC, Niederrhein University of Applied Sciences, Krefeld, Germany

**Correction to: Anal Bioanal Chem**

10.1007/s00216-025-05779-2

The authors regret that the reported concentration of the catalyst sulfuric acid was incorrect. The correct concentration was 89 mol/m^3^. In consequence, the space-time yields and the effective rate constants of the compared batch reactors were recalculated according to equation 3 and compared with the values for batch reactors [39]. Fig. 4 and Tab. 1, therefore, were incorrect.

Old version of Figure 4:
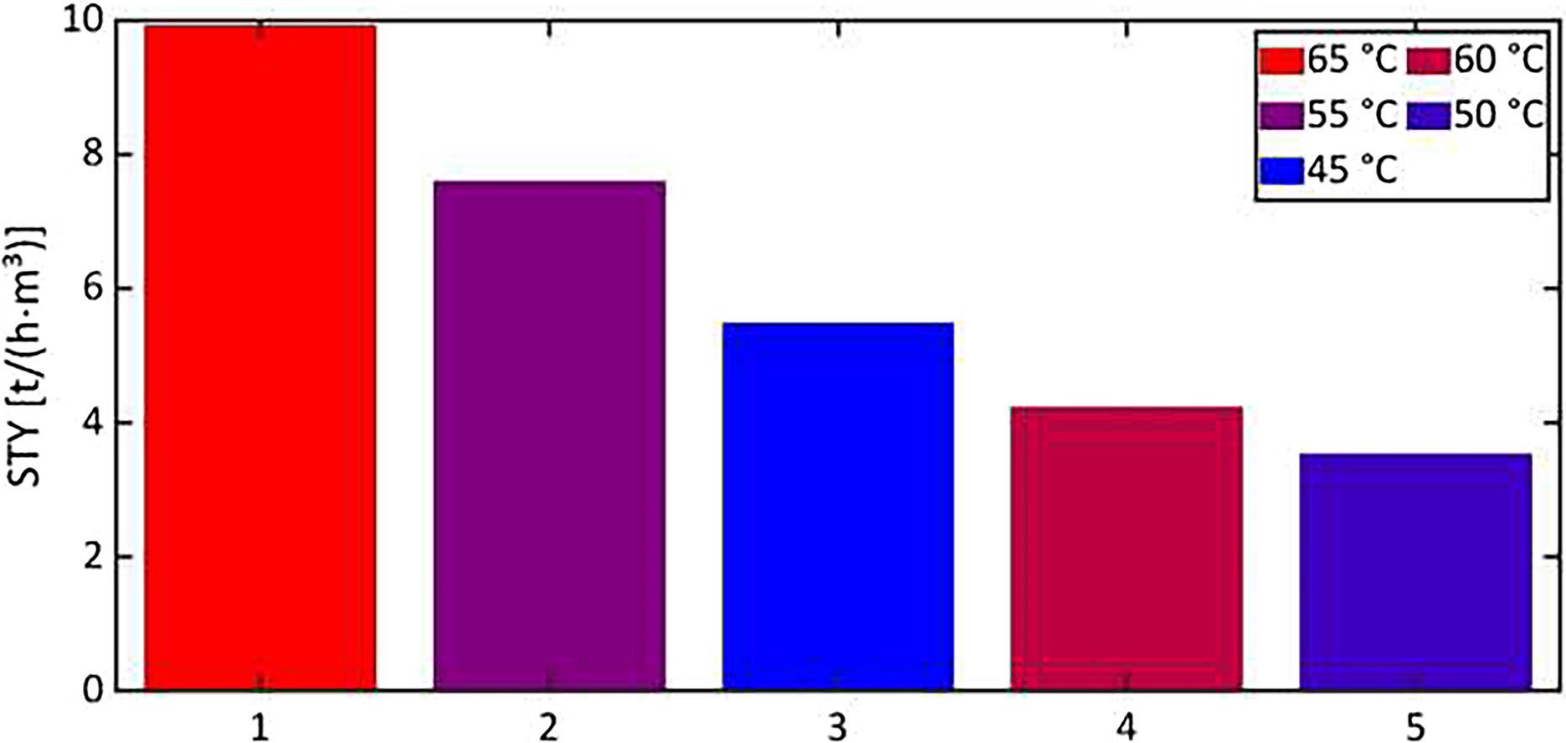


**Fig 4:** Space–time yields of the microreactor system (1–3), a 50-ml batch reactor (4), and a 500-ml batch reactor (5) with 60 mol/m^3^ catalyst (1–4) and with 63.3 mol/m^3^ catalyst (5) at different temperatures. The values for 4 and 5 were taken from [38] and [39], calculated with the total reactor volume used for production and without change-over times

New version of Figure 4:
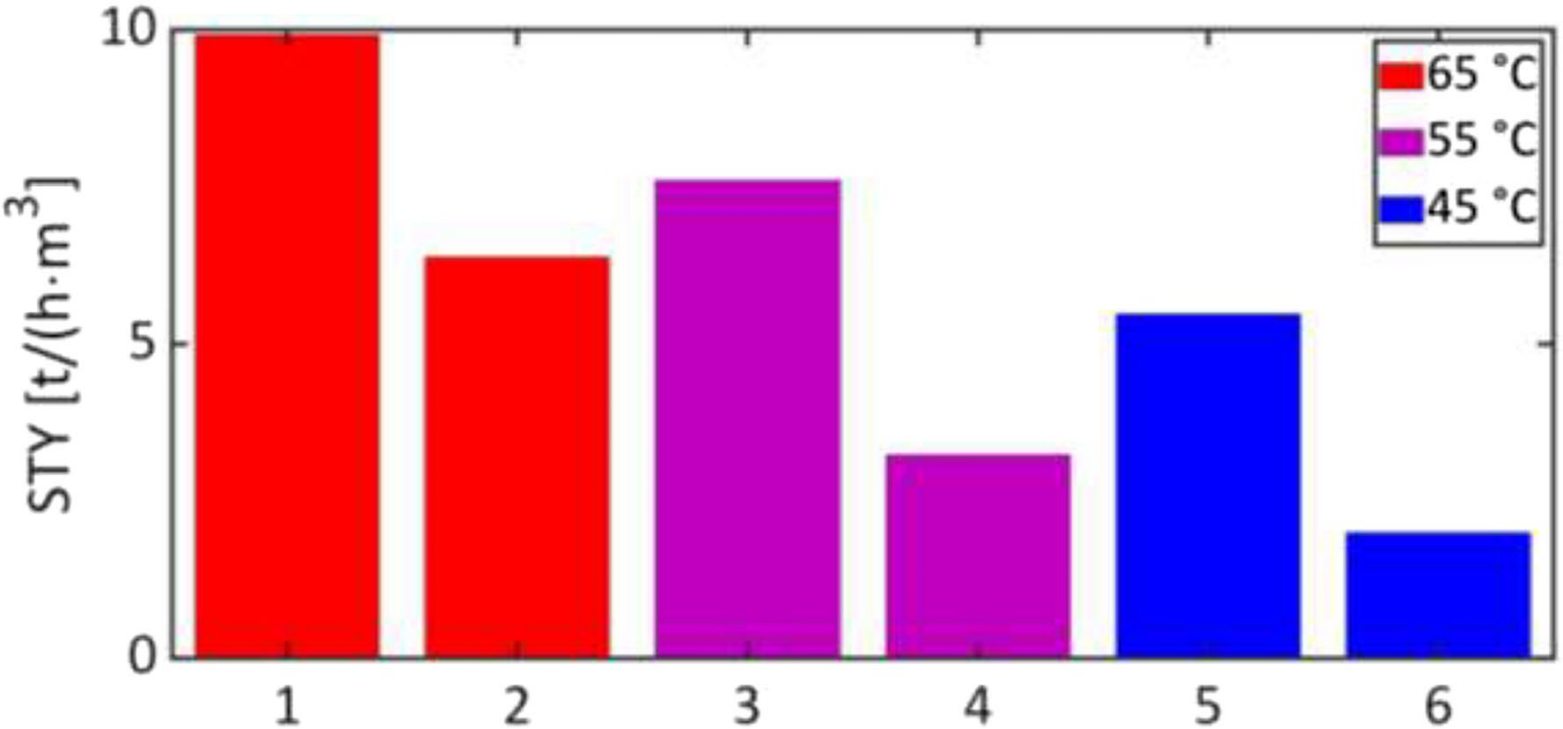


**Fig. 4:** Space-time yields of the microreactor system (1, 3, and 5) and a 500 ml batch reactor (2, 4, and 6) with 89 mol/m^3^ catalyst at different temperatures. The values of the 500 ml batch reactor were taken from [39], calculated with the total reactor volume used for production and without change-over times

Old version of Table 1:

**Table 1** Effective reaction rate constants of the esterification of methanol and acetic acid, based on the microreactor system (MRS) [38, 39]

**Table:** 

Reactor size	Temperature	Catalyst concentration^a^	k_eff_^b^
MRS	50 °C	60	0.02034
500 ml	50 °C	63	0.00712 [39]
MRS	60 °C	60	0.03073
500 ml	60 °C	60	0.02385 [38]

^a^ [mol/m^3^]; ^b^ [l/(min∙mol)]

New version of Table 1:

**Tab. 1** Effective reaction rate constants of the esterification of methanol and acetic acid, based on the microreactor system (MRS) and [39]

**Table:** 

Reactor size	Temperature	Catalyst concentration^a^	k_eff_^b^
MRS	55 °C	89	0.02508
500 ml	55 °C	89	0.01110 [39]
MRS	65 °C	89	0.03742
500 ml	65 °C	89	0.02190 [39]

^a^ [mol/m^3^]; ^b^ [l/(min∙mol)]

In ﻿addition, in section “Conversion and space–time yield” the third paragraph was incorrectly stating:

Figure 4 illustrates that the microreactor assembly produced up to twice the amount of methyl acetate in comparison to conventional batch reactors, even when operated at identical temperatures and catalyst concentrations. The change-over times of the batch reactors were excluded from the calculation, which yields the best-case scenario for the batch reactors. A more rigorous comparison between the microreactor system and the batch reactors is achieved by taking the effective rate constants at different temperatures into account (cf. Table 1). The rate constants of the microreactor system were found between 1.2 and 2.9 times higher than those of the batch reactors.

The correct statement is:

Figure 4 illustrates that the microreactor assembly produced up to 2.8 times the amount of methyl acetate in comparison to conventional batch reactors, even when operated at identical temperatures and catalyst concentrations. The change-over times of the batch reactors were excluded from the calculation, which yields the best-case scenario for the batch reactors. A more rigorous comparison between the microreactor system and the batch reactors is achieved by taking the effective rate constants at different temperatures into account (cf. Table 1). The rate constants of the microreactor system were found between 1.7 and 2.3 times higher than those of the batch reactors.

The authors confirm that these corrections do not impact the discussion and conclusions of the published paper.

The original article has been corrected.

