# Supplementary material for: A process monitoring microreactor assembly for real-time reaction analysis using inline near-infrared spectroscopy and chemometrics
Source: Anal Bioanal Chem. 2025 Feb 7;417(8):1431–9. doi: 10.1007/s00216-025-05779-2 (PMC11876253; doi:10.1007/s00216-025-05779-2)
Supplement: Supplementary file 1 — Supplementary file1 (DOCX 1078 KB) [file 216_2025_5779_MOESM1_ESM.docx]

Article Title:
A Process Monitoring Microreactor Assembly for Real-Time Reaction Analysis Using Inline Near-Infrared Spectroscopy and Chemometrics

Journal Name:
Analytical and Bioanalytical Chemistry

Author Names and Affiliations:
Lukas Mahler^1,2^, Pascal Desel^1,2^, Marcel Sladkov^2^, Andreas Roppertz^2^, Christian Mayer^1^, Martin Jaeger^2^

^1^ University Duisburg-Essen, Department of Physical Chemistry, Universitätsstraße, Essen, D
^2^ Niederrhein University of Applied Sciences, Department of Chemistry and ILOC, Frankenring, Krefeld, D

E-Mail Address of the Corresponding Author:
martin.jaeger@hs-niederrhein.de

| 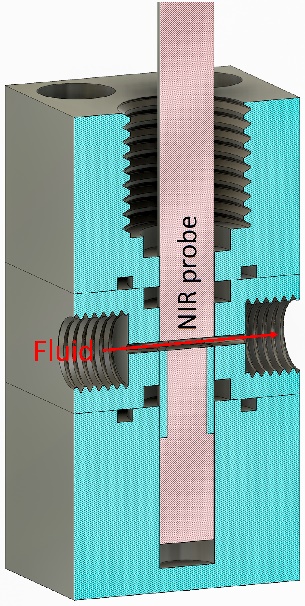 | 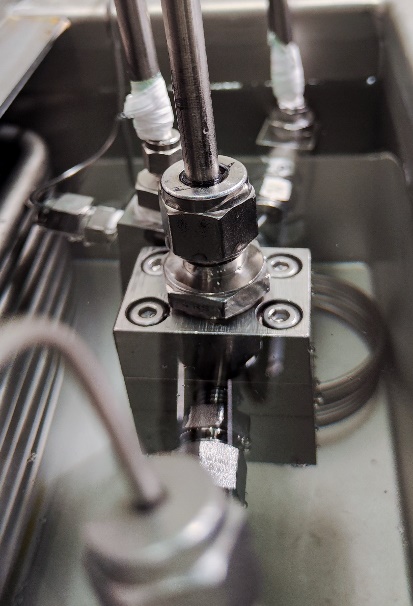 | 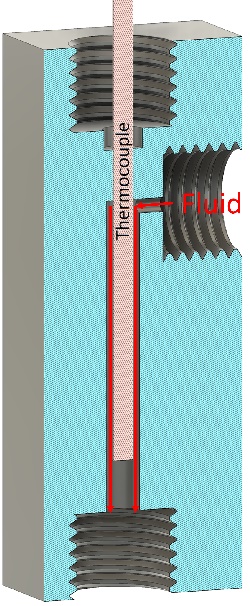 |
| --- | --- | --- |

**Fig. S1** Stainless steel modules for low dead-volume embedding of the NIR probe in cross-section (left) and installed in the microreactor system (middle) and of the resistance temperature devices in cross-section (right)

**Tab. S1** Concentration ranges of the four components for the calibration of the NIR spectrometer determined by off-line gas chromatography

| Component | Concentration range^a^ |
| --- | --- |
| Acetic acid | 5.54 – 10.01 |
| Methanol | 5.54 – 10.01 |
| Methyl acetate | 0.22 – 4.70 |
| Water | 0.22 – 4.70 |

^a^ [mol/l]


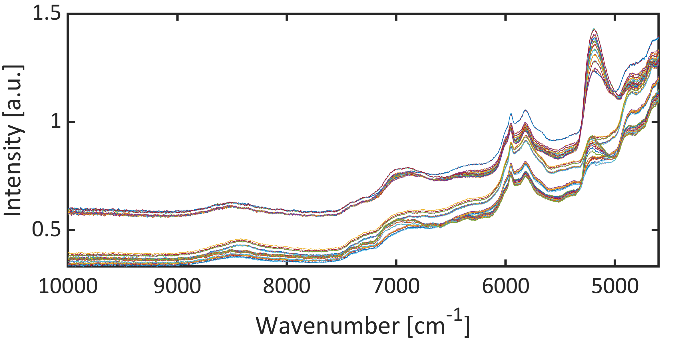

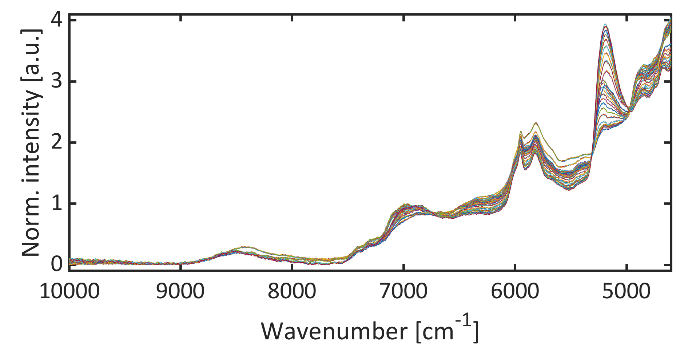


**Fig. S2** NIR spectra of the calibration samples before (left) and after (right) preprocessing via 0^th^ order Savitzky-Golay smoothing with a filter width of 3 points, standard normal variate transformation, and baseline correction


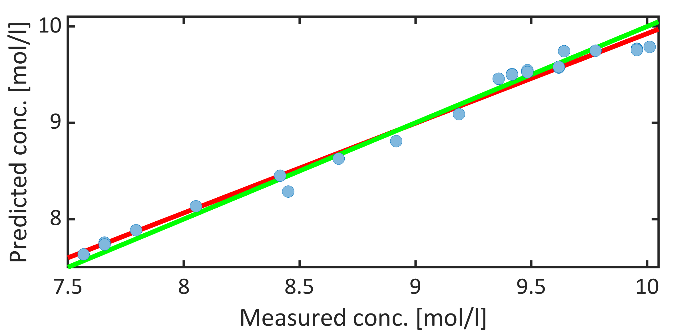

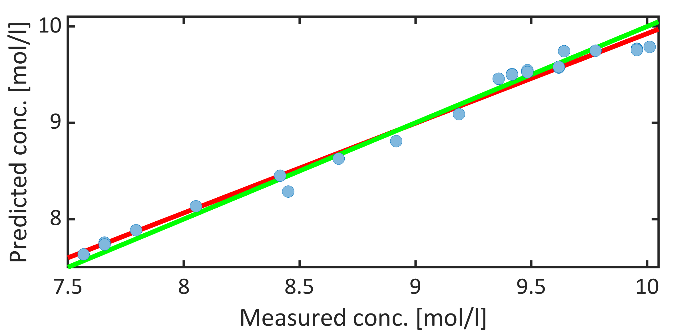


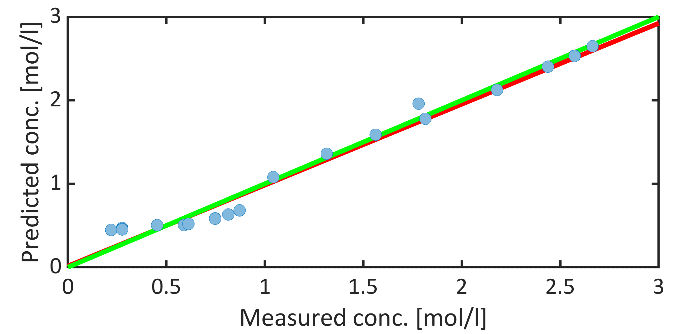

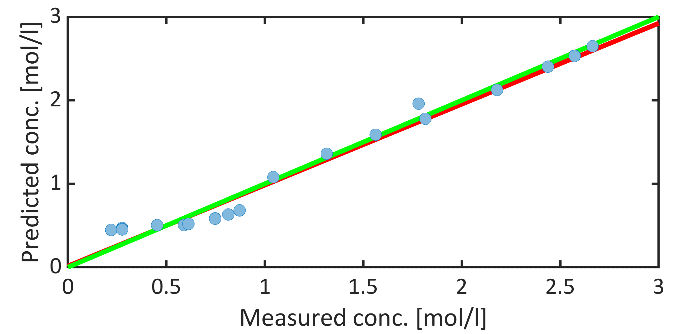


**Fig. S3** Measured versus predicted concentrations (circles) of acetic acid (upper left), methanol (upper right), methyl acetate (bottom left), and water (bottom right) of the validation data sets of the partial least squares regressions based on NIR spectra together with their mathematical fits (red line) and the theoretically expected angle bisector (green line)

**Tab. S2** Number of latent variables, explained variances of the spectral data set X and the concentration data set Y, together with the RMSEPs of the NIR models corresponding to the four components involved in the esterification

|  | Acetic acid | Methanol | Methyl acetate | Water |
| --- | --- | --- | --- | --- |
| Number of latent variables | 3 | 3 | 3 | 3 |
| Explained variance of data set X^a^ | 99.99 | 99.99 | 99.99 | 99.99 |
| Explained variance of data set Y^a^ | 99.99 | 99.99 | 99.94 | 99.94 |
| RMSEP^b^ | 0.114 | 0.114 | 0.138 | 0.138 |

^a^ [%], ^b^ [mol/l]

**Eq. S1** Root mean square error of the prediction (RMSEP) with $y_{i}$ as the measured concentration, $\hat{y}_{i}$ as the calculated concentration and $n$ as the number of samples

$$RMSEP=\sqrt{\frac{\sum_{i=1}^{n} \left( y_{i}-\hat{y}_{i} \right)^{2}}{n}}$$

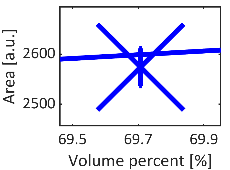

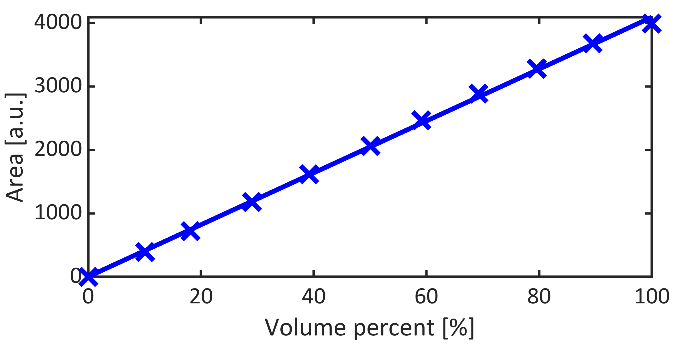

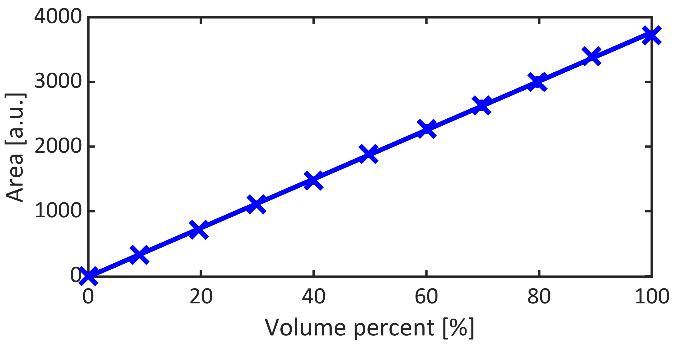


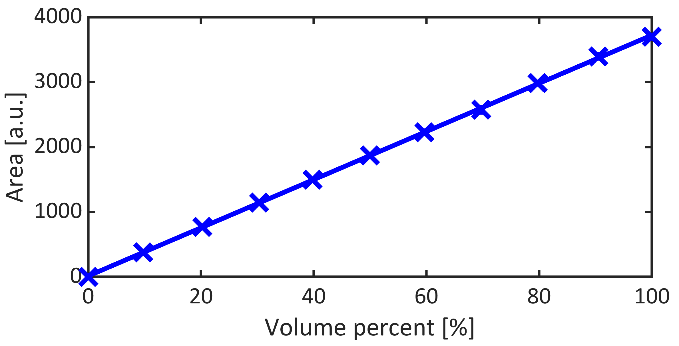

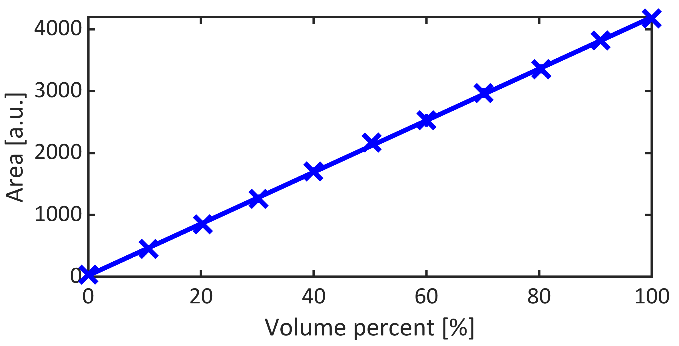


**Fig. S4** Gas chromatography calibration plots of acetic acid (upper left), methanol (upper right), methyl acetate (bottom left), and water (bottom right) together with their error bars. A zoomed in example of an error bar is shown in the calibration plot of methyl acetate.

**Tab. S3** Calibration formulas and regression coefficients of the gas chromatography calibrations of each of the four components

| Component | Calibration formula | Regression coefficient |
| --- | --- | --- |
| Acetic acid | $y=4088.5x+2.2281$ | 0.9991 |
| Methanol | $y=3778.5x+12.552$ | 0.9996 |
| Methyl acetate | $y=3709.2x+13.406$ | 0.9999 |
| Water | $y=4174.2x+20.964$ | 0.9997 |

where $y$ is the area of the corresponding peak and $x$ is the volume percentage of the component.


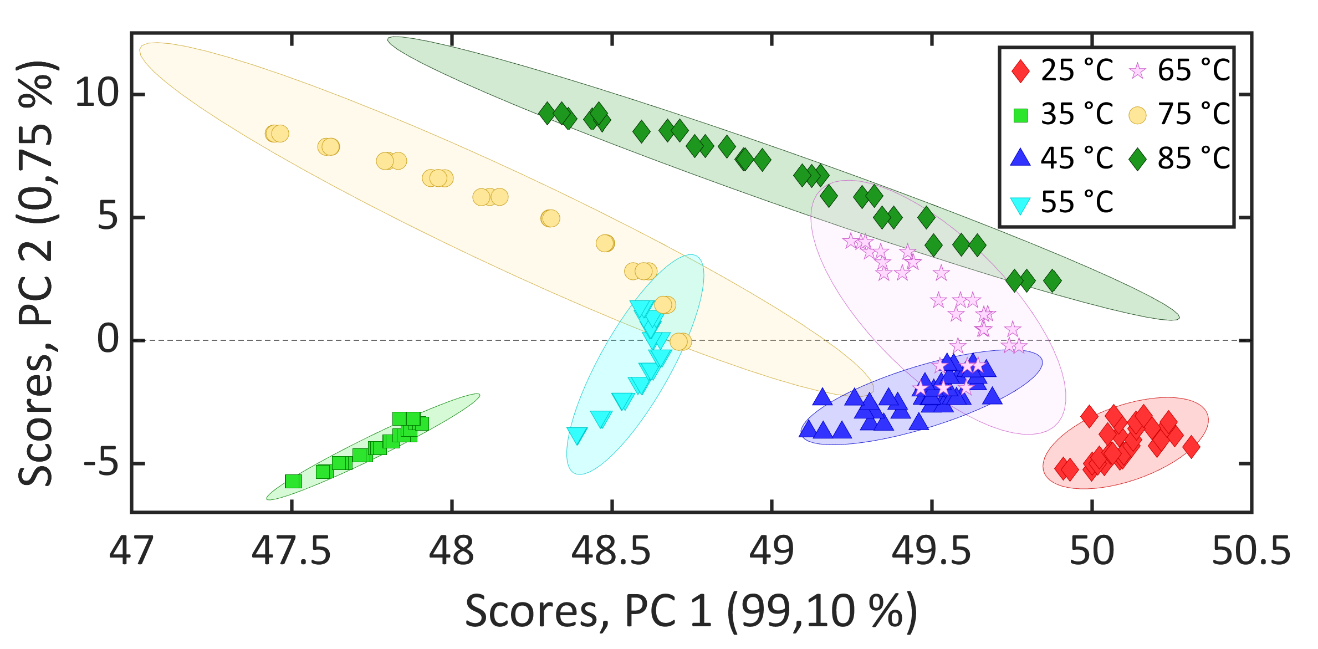

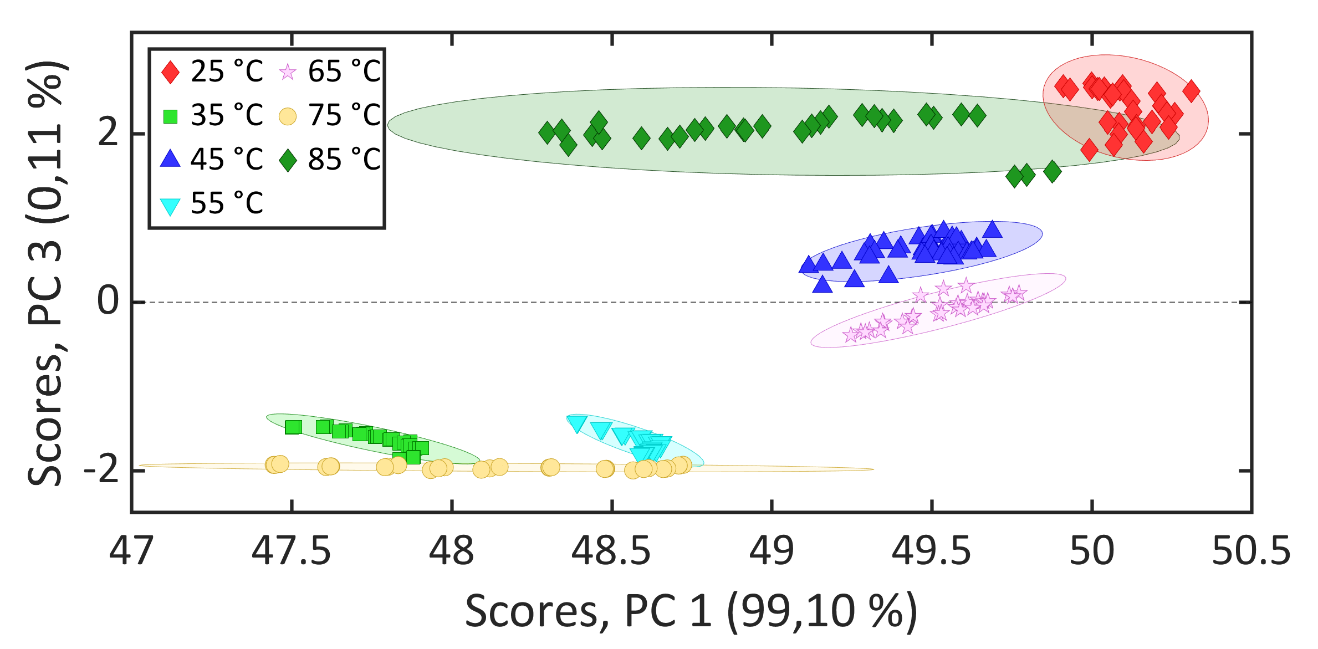

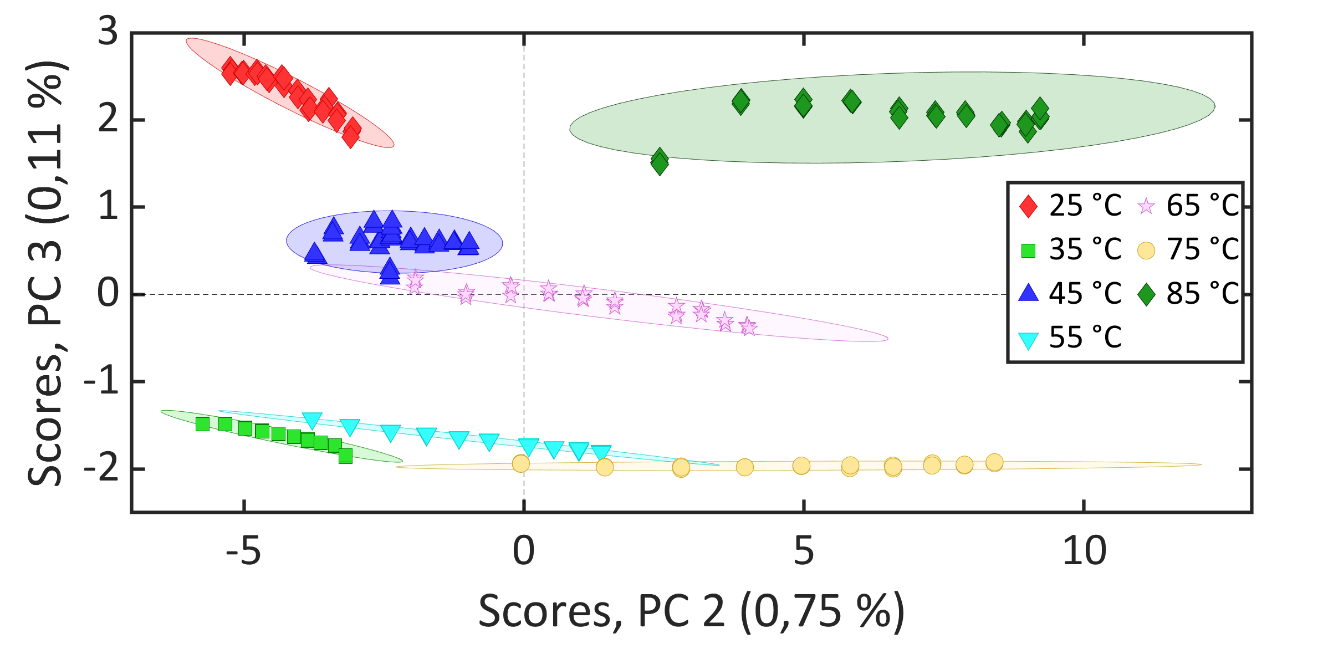


**Fig. S5** Scores plots of PC 2 vs. PC 3 (bottom), PC 1 vs. PC 3 (middle), and PC 1 vs. PC 2 (top) of the 222 NIR spectra categorized in the seven different reaction temperatures with their individual confidence ellipses


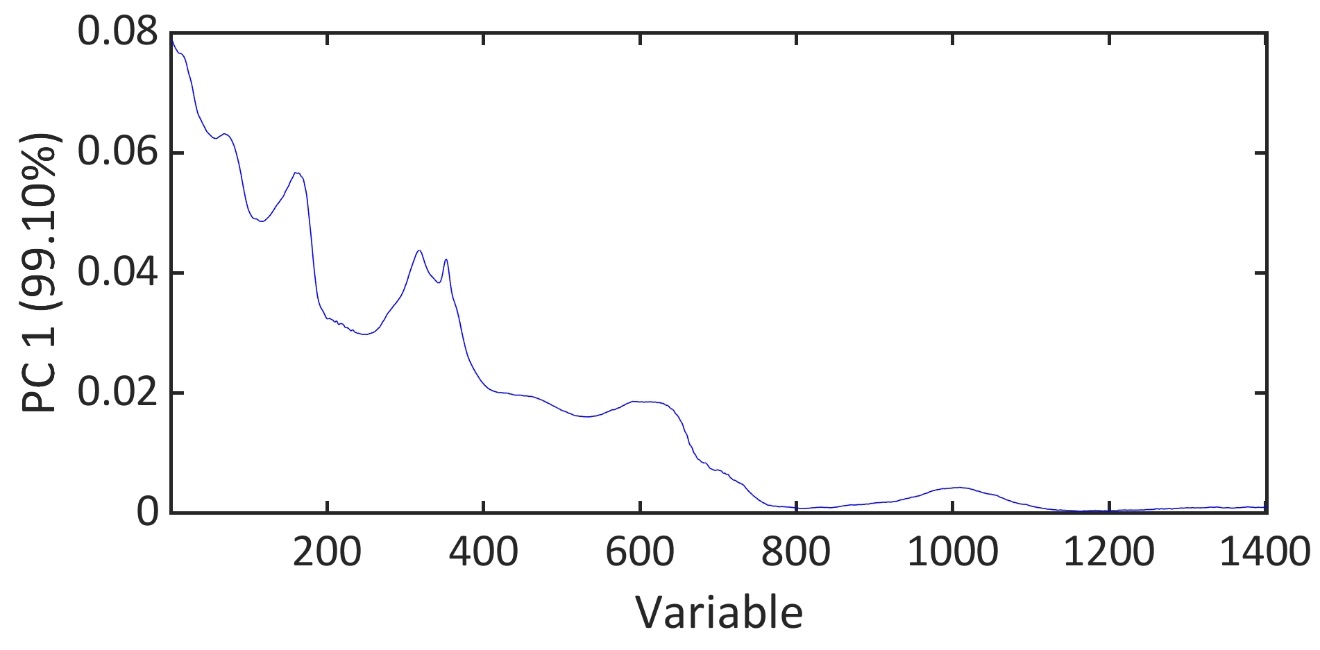

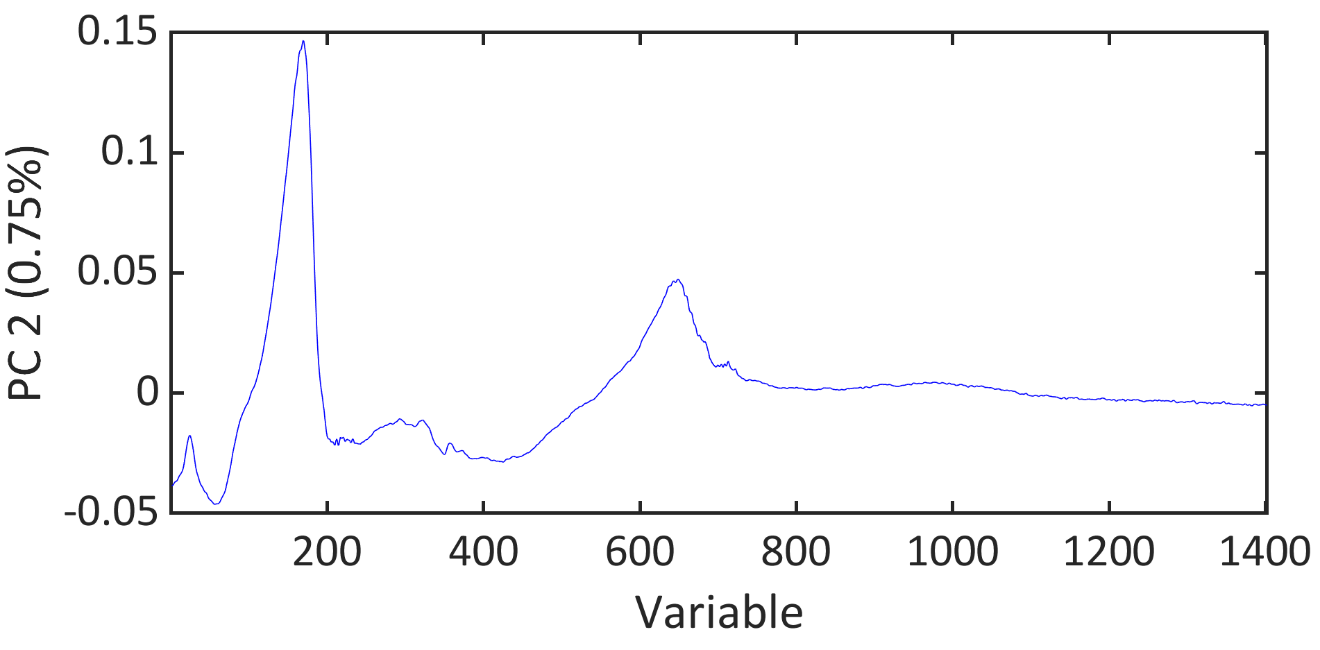

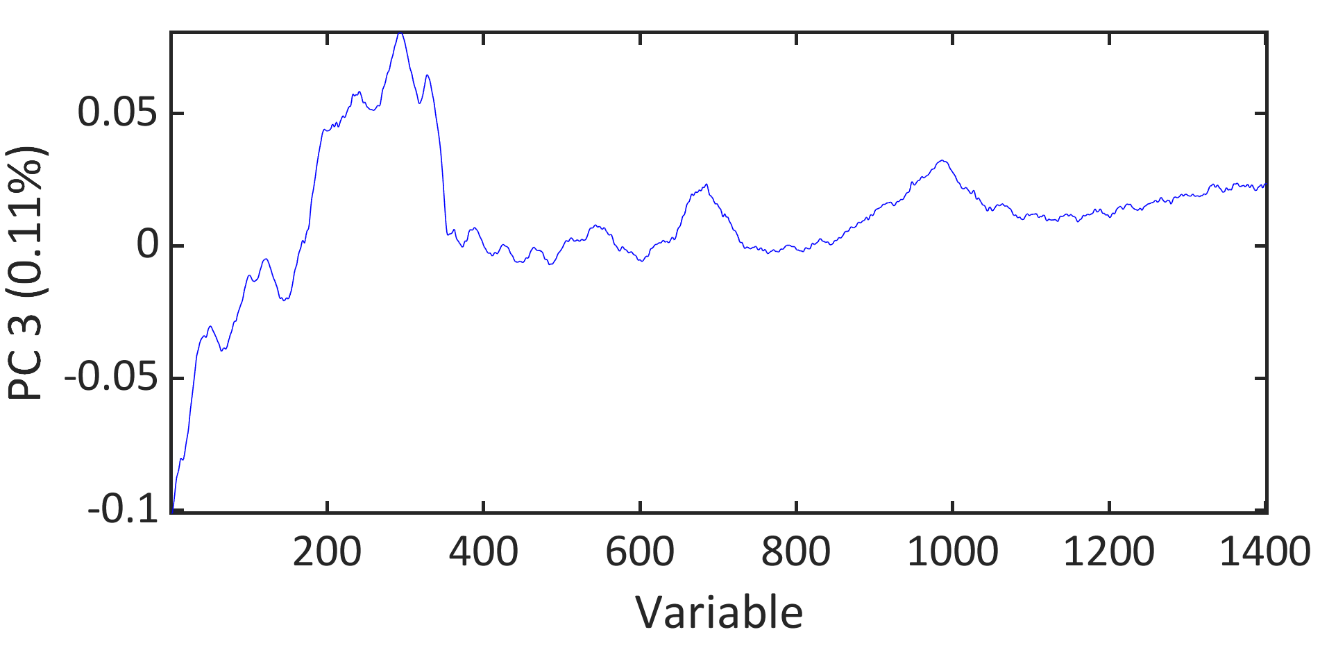


**Fig. S6** Loadings of PC 3 (bottom), PC 2 (middle), and PC 1 (top) of the principle component analysis of the 222 NIR spectra


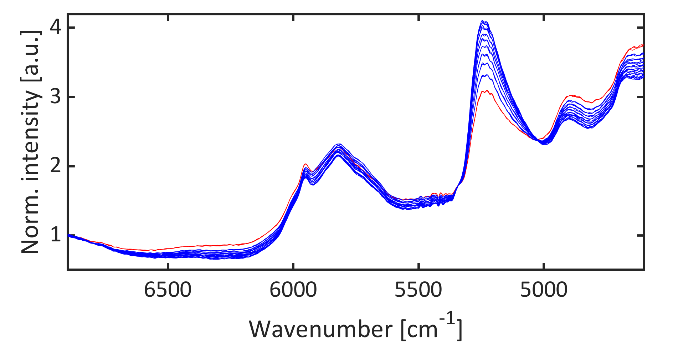

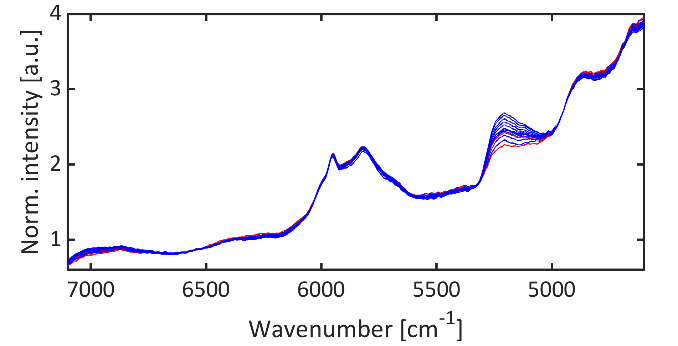


**Fig. S7** NIR spectra of the reaction mixture with reaction temperatures of 85 °C (left) and 45 °C (right) with anomalous spectra in red
